# Analysis of miRNAs Involved in Mouse Heart Injury Upon Coxsackievirus A2 Infection

**DOI:** 10.3389/fcimb.2022.765445

**Published:** 2022-01-28

**Authors:** Zhaoke Wu, Shenshen Zhu, Juanfeng Qian, Yanmin Hu, Wangquan Ji, Dong Li, Peiyu Zhu, Ruonan Liang, Yuefei Jin

**Affiliations:** ^1^Department of Gerontology, The Second Affiliated Hospital of Zhengzhou University, Zhengzhou, China; ^2^Department of Epidemiology, College of Public Health, Zhengzhou University, Zhengzhou, China

**Keywords:** hand, foot, and mouth disease, Coxsackievirus A2, miRNAs, RNA-seq, molecular mechanism

## Abstract

Coxsackievirus A2 (CVA2) has recently been constantly detected, and is associated with viral myocarditis in children. Our previous study demonstrated that CVA2 led to heart damage in a neonatal murine model. However, the molecular mechanism of heart injury caused by CVA2 remains largely unknown. Emerging evidence suggests the significant functions of miRNAs in Coxsackievirus infection. To investigate potential miRNAs involved in heart injury caused by CVA2, our study, for the first time, conducted a RNA-seq *in vivo* employing infected mice hearts. In total, 87, 101 and 76 differentially expressed miRNAs were identified at 3 days post infection (dpi), 7 dpi and 7 dpi vs 3 dpi. Importantly, above 3 comparison strategies shared 34 differentially expressed miRNAs. These results were confirmed by quantitative PCR (qPCR). Next, we did GO, KEGG, and miRNA-mRNA integrated analysis of differential miRNAs. The dual-luciferase reporter assay confirmed the miRNA-mRNA pairs. To further confirm the above enriched pathways and processes, we did Western blotting and immunofluorescence staining. Our results suggest that inflammatory responses, T cell activation, apoptosis, autophagy, antiviral immunity, NK cell infiltration, and the disruption of tight junctions are involved in the pathogenesis of heart injury caused by CVA2. The dysregulated miRNAs and pathways recognized in the current study can improve the understanding of the intricate interactions between CVA2 and the heart injury, opening a novel avenue for the future study of CVA2 pathogenesis.

## Introduction

Hand, foot, and mouth disease (HFMD) and herpangina are two principal childhood infectious diseases. Coxsackievirus belongs to a member of the genus *Enterovirus* (EV) and the family *Picornaviridae*. Recently, Coxsackievirus A2 (CVA2) has been recognized as a considerable etiological agent in the pathogen spectrum of HFMD and herpangina worldwide (Chansaenroj et al., 2017; Ai et al., 2021). In 2012, 4 young children were reported with serious upper respiratory illness due to CVA2 infection in Hong Kong, two of whom died (Yip et al., 2013). In 2009~2013, it was reported that CVA2 emerged as one of the most leading types of the 12 circulating serotypes of EVs arousing HFMD in Jinan, Shandong Province (Guan et al., 2015). In 2019, an epidemic of herpangina occurred in Zunyi, Guizhou Province owing to CVA2 infection (Ai et al., 2021). Infection predominantly by CVA2 caused herpangina in 2015 in Thailand (Chansaenroj et al., 2017). CVA2 is reported all over the world, especially in Asia-Pacific region (China (Yip et al., 2013), Vietnam (Hoa-Tran et al., 2020), Thailand (Chansaenroj et al., 2017), and Korea (Park et al., 2012)) and France (Molet et al., 2016), Australia (Cobbin et al., 2018)), posing a great challenge for global public health.

Generally, HFMD and herpangina are self-limiting diseases, but some infections can develop into severe complications including encephalitis, meningitis, myocarditis, cardiopulmonary failure and can occasionally lead to death (Jiang et al., 2012; Cai et al., 2019; Hoang et al., 2019; Qiu et al., 2019). Previous studies reported myocarditis in CVA2 infections (Bendig et al., 2001; Yip et al., 2013). In July 1999, a previously healthy 10-year-old girl collapsed and died whilst running. Postmortem histologic examination showed myocarditis with lymphocytes scattered throughout the myocardium with occasional focal collections of lymphocytes associated with fibre necrosis. Nucleotide sequence analysis suggested an EV resembling CVA2 (Bendig et al., 2001). A case of sudden death in a 4-year-old boy with post-mortem findings of CVA2 replication in tissue samples of heart, spleen, lung, and rectum, and nasopharyngeal and rectal swab specimens (Yip et al., 2013). Additionally, our previous animal model of CVA2 showed heart injury characterized with leukocyte infiltration and myocardial rupture. We also detected viral replication in heart tissues of infected mice (Ji et al., 2021). However, the mechanism of CVA2-associated heart injury remains mostly unclear.

MicroRNAs (miRNAs) are a class of small endogenous RNAs regulating gene-expression post-transcriptionally in both normal physiological circumstances and in disease conditions. MiRNA expression profiling is gaining importance since miRNAs, as important moderators in networks of gene expression, can affect a lot of biological processes and also display promise as novel diagnostic and therapeutic approaches for human disease (Pritchard et al., 2012; Lu and Rothenberg, 2018). Previous studies have demonstrated that abnormal expression and functioning of miRNA is linked to heart injury induced by viral infection (Zhang et al., 2020). Coxsackievirus B (CVB) is known as the primary cause of human myocarditis and dilated cardiomyopathy. Up-regulation of miR-217 and miR-543 has been reported in the peripheral blood of patients with viral myocarditis caused by CVB3, and these miRNAs further facilitate myocardial injury by targeting the Sirtuin1 lysine deacetylase (SIRT1)/AMPK/nuclear factor−κB (NF−κB) pathway (Xia et al., 2020). The miR-221/-222 cluster is increased during CVB3-induced acute viral myocarditis, which in turn regulates viral replication and inflammation (Corsten et al., 2015). CVB3-induced miR-20b increases the expression of anti-apoptosis proteins Bcl-2 and Bcl-xL, and further promotes viral replication (Xu et al., 2017). The up-regulation of miR-223 protects the mice against CVB3-induced myocardial injuries (Gou et al., 2018). Additionally, EV-induced miR-146a contributes viral pathogenesis by suppressing interferon (IFN) production (Ho et al., 2014). Yang et al. found that weaken miR-155-5p promotes EV71 replication through suppression of type I IFN response (Yang et al., 2020).

In the present study, we established a neonatal murine model to uncover the molecular mechanisms of miRNAs in heart damage caused by CVA2 infection.

## Materials and Methods

### Cells and Viruses

Human rhabdomoma (RD), and HEK293 cells were cultured in DMEM (Thermo Fisher Scientific Inc., MA, USA) supplemented with 10% fetal bovine serum (FBS, Thermo Fisher Scientific Inc., MA, USA) and incubated at 37 °C with 5% CO_2_. The CVA2 strain HN202009 (the accession number is MT992622) was applied for mouse infection. The viral titers of tissues were measured with TCID_50_ assay according to Reed’s and Muench’s method. CVA2 stocks were undergone three freeze-thaw cycles, clarified by centrifugation at 4,000×*g* for 10 min at 4°C, filtered with a 0.22 µm filter, and deposited at −80°C. In this study, the titer of CVA2 stocks is 2.45 × 10^7^ TCID_50_/mL.

### Animal Model and Ethics Statement

BALB/c mice used in this research were purchased from the Henan Experimental Animal Center, and all animals were housed in a specific pathogen-free facility of the College of Public Health of Zhengzhou University on a 12 h light/dark cycle and allowed free access to food and water. As described previously (Ji et al., 2021), we established a neonatal mouse model of CVA2 infection based on five-day-old BALB/c mice inoculated with a lethal dose of CVA2 strain (10^4^TCID_50_/mouse, accesses number HN202009) *via* intramuscular (i.m.) route. Control and CVA2-infected mice were sacrificed at 3 (n=3/group) or/and 7 days post infection (dpi) (n=3/group), respectively. The heart tissues of all mice were either performed 4% paraformaldehyde-fixation, paraffin-embedding for histopathological analysis or stored at −80°C for RNA isolation, RNA-seq, and quantitative PCR (qPCR). The study presented in this manuscript was authorized by the Life Science Ethics Review Committee of Zhengzhou University with a permission number of ZZUIRB2020-29.

### Histopathological Analysis

At 3 and 7 dpi, heart samples were collected, fixed by 4% paraformaldehyde, embedded in paraffin, sectioned for 5 μm thickness, and finally stained with hematoxylin-eosin (H&E). Apoptosis was determined in heart slices using the terminal transferase-mediated DNA nick end labeling (TUNEL) staining (Wuhan Servicebio Technology Co., Ltd., Wuhan, China). Red staining indicates TUNEL-positive cells. The localization of CD11b in heart slices was conducted by immunofluorescence (IF) staining. As described previously (Jin et al., 2021), IF staining and scanning technical service were commissioned by Servicebio Biotech Co., Ltd. CD11b positive cells were calculated by Image J software.

### RNA Extraction, miRNAs Library Construction, and Sequencing

Total RNA was extracted from mice hearts using TRIzol (Thermo Fisher Scientific Inc., MA, USA). A total amount of 2.5 ng RNA/mice heart was used as input material for the RNA sample preparations. The RNA molecules with a size range of 18-30 nt were then enriched by polyacrylamide gel electrophoresis (PAGE). The 3’ adapters were added, and the 36-44 nt RNAs were enriched. The 5’ adapters were then also ligated to the RNAs. The ligation products were reverse transcribed by PCR amplification, and the 140-160 bp purified PCR products were enriched to generate a cDNA library with TruSeq Small RNA Sample Prep Kits (Illumina, San Diego, USA). and sequenced using Illumina HiSeqTM 2500 by Gene Denovo Biotechnology Co. (Guangzhou, China). Sequencing was performed using an Illumina Hiseq2000/2500 instrument. This part of the experiment was commissioned by the TaoHarmony Bio (TaoHarmony Biotechnology Co. Ltd, Hangzhou, China).

### MiRNA Expression Profile and PCA Analysis

For both known and novel miRNAs, the miRNA expression level was calculated and normalized by transcripts per million (TPM). The equation was as follows: TPM = Actual miRNA counts∗10^6^/Total counts of clean tags. The principal component analysis (PCA) was conducted using TPM of all miRNAs (TPM > 1, n = 224) by R package (http://www.r-project.org/). Heatmaps were created by using R scripts.

### Analysis of Differentially Expressed MiRNAs

Firstly, the expression levels of miRNAs at different stages were filtered by TPM > 1. Next, differential miRNAs between region pairs were carried out using the DESeq R package (version 1.10.1). DESeq2 (Love et al., 2014), like edgeR, can provide computational pipelines for determining differential expression in digital miRNA expression data using a model on account of the negative binomial distribution. Finally, in the differential miRNAs detection process, p-value <0.05 was used as the screening standard. Heatmaps were created by using own R scripts. Differential miRNAs with p-value <0.05 and the value of |log2 (fold change)| > 1 were used to draw the volcano map with our own scripts.

### Target Gene Prediction and Functional Enrichment Analysis

Potential miRNA targets of all differentially expressed miRNAs at different stages were predicted with the online software miRWalk (http://mirwalk.umm.uni-heidelberg.de/) and miRDB (http://mirdb.org/) together. The target genes predicted by these two softwares were screened according to the scoring standards of each software. The Gene Ontology (GO) analysis characterizes gene with a biological function, such as molecular function (MF), biological process (BP), or cellular component (CC) (Ashburner et al., 2000). The Kyoto Encyclopedia of Genes and Genomes (KEGG) analysis offers annotation information of signal transduction and disease pathways for genes, providing a basis for gene function and pathway research (Young et al., 2010). We mapped all of the candidate targets to the GO database (http://www.geneontology.org/) and KEGG (https://www.genome.jp/kegg/) using the R package clusterProfiler (Yu et al., 2012). The terms or pathways with p-adjusted value <0.05 were regarded as significantly enriched pathways. We applied Cytoscape (version 3.5.1) to construct interaction networks between differential co-expressed miRNAs and target genes. In this fashion, we were capable of visualizing genes that are being targeted by dysregulated miRNAs.

### Total RNA Isolation and qPCR

Total RNA of mice heart tissues was extracted by using 1 mL TRIzol reagent (Thermo Fisher Scientific Inc., MA, USA) on the basis of manufacturer’s methods. The quality of the purified RNA was determined by 1% agarose gels, and the concentration was determined by measuring the absorption ratio at 260/280 nm using a NanoDrop ND-2000 (Thermo Fisher Scientific Inc., MA, USA). 1 µg of total RNA was reverse transcribed into cDNA with the PrimeScript RT reagent kit (TaKaRa Biotechnology (Dalian) Co., Ltd, Dalian, China) and qPCR was run with TB Green^®^ Premix Ex Taq™ (TaKaRa Biotechnology Co., Ltd, Dalian, China) according to standard protocol. The transcription level of CVA2 VP1 was performed with the following primers: VP1 primer (forward), 5’-TCAGTCCCATTCATGTCGCC-3’; VP1 primer (reverse), 5’-AATGCGTTGTTGGGGCATTG-3’; mouse β-actin (forward), 5’-GTGCTATGTTGCTCTAGACTTCG-3’; mouse β-actin (reverse), 5’-ATGCCACAGGATTCCATACC-3’. For miRNAs cDNA synthesis, we used the stem-loop method with miRNA cDNA Synthesis Kit (Vazyme Biotech Co.,Ltd, Nanjing, China) according to the manufacturer protocol. QPCR was conducted using a miRNA qPCR Assay Kit (Vazyme Biotech Co.,Ltd, Nanjing, China). U6 was used as reference gene for normalization. Expression levels of miRNAs were quantified as relative values vs that of U6 using the 2^-ΔΔCt^ method. Primers used in the present study were list in **Table 1**.

**Table 1:** Primers used in this study.

Target Gene	Forward primer (5’ to 3’)	Reverse primer (5’ to 3’)	miRBase ID
mmu-miR-29a-3p	CGCGTAGCACCATCTGAAAT	AGTGCAGGGTCCGAGGTATT	MIMAT0000535
mmu-miR-29c-3p	CGCGTAGCACCATTTGAAAT	AGTGCAGGGTCCGAGGTATT	MIMAT0000536
mmu-miR-511-3p	GCGCGAATGTGTAGCAAAAGA	AGTGCAGGGTCCGAGGTATT	MIMAT0017281
mmu-miR-208a-3p	CGCGATAAGACGAGCAAAAA	AGTGCAGGGTCCGAGGTATT	MIMAT0000520
mmu-miR-22-5p	CGCGAGTTCTTCAGTGGCAA	AGTGCAGGGTCCGAGGTATT	MIMAT0004629
mmu-miR-130a-3p	CGCGCAGTGCAATGTTAAAA	AGTGCAGGGTCCGAGGTATT	MIMAT0000141
mmu-miR-3072-3p	CGTGCCCCCTCCAGGAAG	AGTGCAGGGTCCGAGGTATT	MIMAT0014853
U6	CTCGCTTCGGCAGCACA	AACGCTTCACGAATTTGCGT	

### Luciferase Reporter Assay

Briefly, a fragment of the 3’-UTR of target gene mRNA containing the putative miRNA binding sequence was cloned and transfected into HEK 293 cells with mimics and negative control. In detail, the 3’-UTR of TRIM37, Eif4e2, and TLR4 part was cloned into the Sac I and Xba I sites in the pmirGLO vector (Promega). The mutant 3’-UTR of TRIM37, Eif4e2, and TLR4 was also cloned into the Sac I and Xba I sites in the pmirGLO vector (Promega). HEK293T cells were seeded in 96 well plates and transiently transfected with luciferase reporter plasmids (pmirGLO-TRIM37 and pmirGLO-mut-TRIM37; pmirGLO-Eif4e2 and pmirGLO-mut-Eif4e2; pmirGLO-TLR4 and pmirGLO-mut-TLR4) and mimics (mmu-miR-130a-3p; mmu-miR-29c-3p; mmu-miR-511-3p) or negative control using Lipofectamine 3000 reagent (Invitrogen). After 48 h, the cells were collected and the activities were detected using the Dual Luciferase Reporter Assay System, according to the manufacturer’s instructions (Promega). The activities were normalized to the Renilla luciferase signal in HEK 293 cells. At least 3 independent repeated experiments were performed for each assay.

### Western Blotting

Total proteins of the heart tissues from control and CVA2-infected mice (7 dpi) were extracted using a protein extraction kit (CWbio Company Ltd., Beijing, China) according to the manufacturer’s instructions. Protein samples were separated by 10% SDS-PAGE and transferred to PVDF membranes. Subsequently, membranes were blocked with 5% non-fat milk powder that was dissolved in PBST (phosphate-buffered saline, pH 7.6, containing 0.05% Tween20) for 1 h at room temperature. After incubation with primary antibodies overnight at 4°C, membranes were washed thrice with PBST and then were incubated with either anti-mouse or rabbit second antibodies for 1 h at room temperature. Finally, the signals on the PVDF membrane were visualized with an ECL enhanced Chemiluminescence Kit (Absin Bioscience, Inc., Shanghai, China).

### Antibodies

The following primary antibodies were used in this study: anti-LC3B (Beyotime Biotechnology, Inc.), anti-CTNI, VE-Cadherin (Proteintech Group, Inc), anti-pERK1/2 (Thr202/Tyr204), pSTAT1 (Ser727), pSAPK/JNK (Thr183/Tyr185), JNK2, ERK1/2, pp38 (Thr180/Tyr182), p38, AKT, pAKT (Thr308), pmTOR (Ser2448) (Cell Signaling Biotechnology, Inc.), anti-CD11b (Abcam Biotechnology, Inc.).

### Statistical Analysis

Statistical analysis was conducted with GraphPad Prism version 8.3 (GraphPad 8.3 Software, San Diego, CA, USA). The results were represented as the mean ± standard deviation (SD). Any differences in survival rates of control and CVA2-infected mice were evaluated with log-rank test. Unpaired Student’s t test or one-way ANOVA was employed to determine the significance of differences between groups. Differences with *p-*value less than 0.05 were considered statistically significant.

## Results

### Establishment of the CVA2-Infected Mouse Model

**Figure 1:**
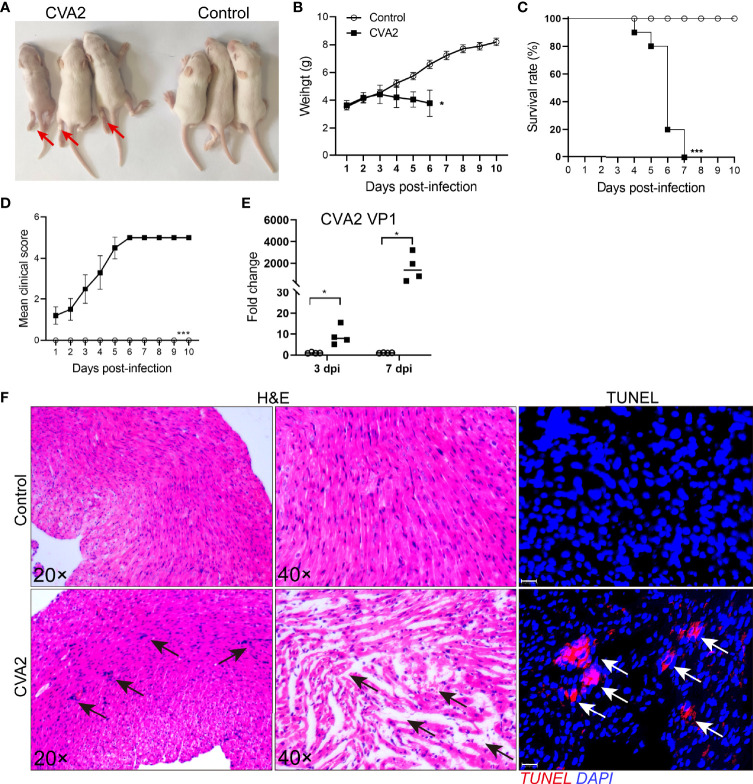
CVA2 infection leads to heart injury in a neonatal mouse model. **(A)** Symptoms of CVA2-infected mice at 5 dpi. The body weight **(B)**, survival rate **(C)**, and clinical scores **(D)** of Control (n=10) and CVA2-infected mice (n=10) were recorded from 1 dpi to 10 dpi. Viral titers **(E)** in heart tissues of mice represented by the fold change of VP1 mRNA were detected by qRT-PCR (n=4). Histopathological changes **(F)** in heart slices of mice were evaluated by H&E staining and TUNEL staining. Red arrows indicate limb paralysis of infected mice. Black arrows indicate inflammatory cell infiltration, myocardial fiber breakage, myocardial interstitial edema, and white arrows indicate apoptotic cells in heart slices of infected mice. *p < 0.05; ***p < 0.001.

Five-day-old BALB/c mice were inoculated with a lethal dose of CVA2 strain *via* intramuscular (i.m.) route in this study. As shown in **Figures 1A, B**, infected mice developed into severity with the manifestations of weight loss, limb paralysis, reduced movement, and dying state. Survival rate (**Figure 1C**) showed that all infected mice died between 4 and 7 dpi, while control mice stayed healthy throughout the experiment (**Figures 1C, D**). We also detected viral replication in heart tissues of infected mice (**Figure 1E**). To confirm the heart injury induced by CVA2, we conducted histopathological examination. As shown in **Figure 1F**, our results showed that inflammatory cell infiltration, myocardial fiber breakage, myocardial interstitial edema in infected hearts, and apoptosis were observed in heart slices of infected mice. Together, our results demonstrated that CVA2 led to heart injury in a neonatal mouse model.

### MiRNA Profile Analysis in Mice Hearts in Response to CVA2 Infection

**Figure 2:**
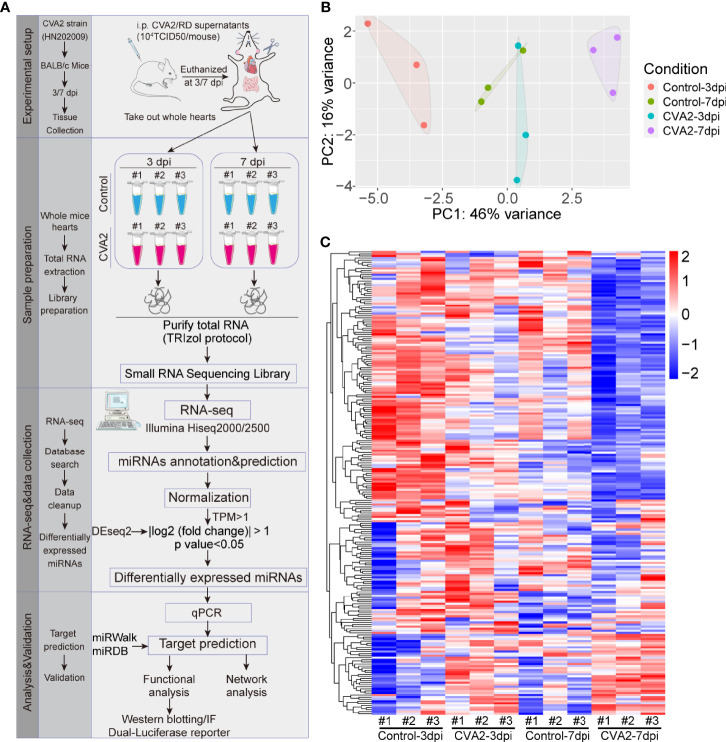
The miRNA expression patterns in heart tissues of CVA2-infected mice. **(A)** Study design of RNA-seq analysis for CVA2-infected heart tissues. A total of 224 miRNAs with TPM > 1 were detected in heart tissues of CVA2-infected mice at 3 and 7 dpi. **(B)** Principal component analysis of the miRNA profile. Biological replicates were generated for each sample, represented by different color points in the figure. **(C)** The expression patterns of miRNAs at 3 dpi, and 7 dpi.

To investigate miRNA expression alterations caused by CVA2 in heart tissues of mice, the miRNA expression profiles were detected at 3, 7 days post-infection (dpi) (**Figure 2A**). Based on the RNA-seq data, there were 224 miRNAs measured in heart tissues of mice with TPM > 1 (**Table S1**). The PCA analysis showed that the miRNAs identified in control and CVA2-infected mice at different stages formed independent clusters (**Figure 2B**). The expression patterns of those miRNAs were displayed in the clustered heatmaps (**Figure 2C**). The expression patterns induced by CVA2 infection at 3 dpi or 7 dpi were quite distinct from the profiles seen in control mice. In addition, difference was also observed in the expression profile of infected mice at 3 dpi and 7 dpi.

**Figure 3:**
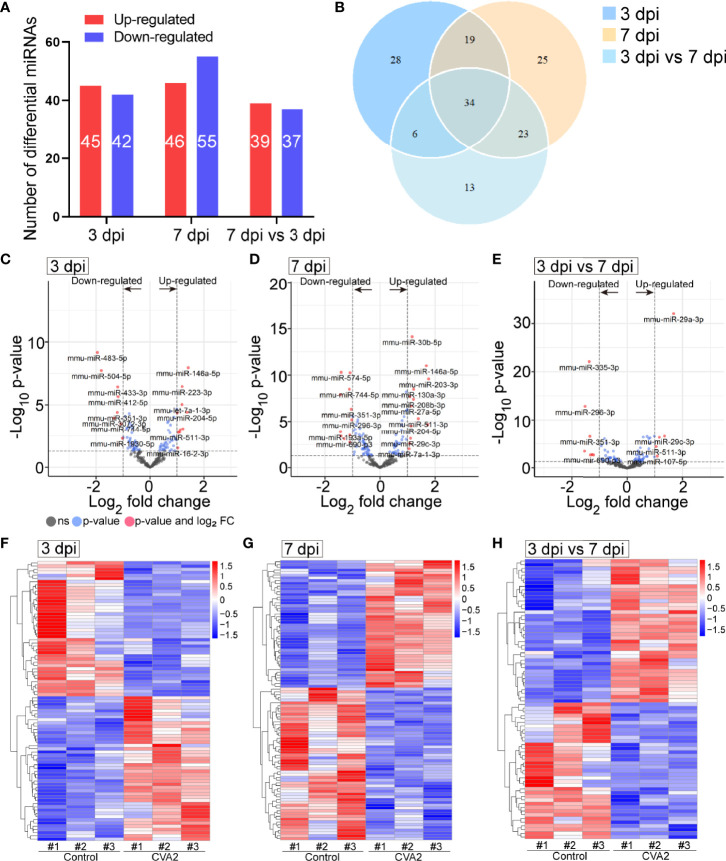
The differentially expressed miRNAs in heart tissues of CVA2-infected mice. **(A)** Statistics of differentially expressed miRNAs among samples. **(B)** Venn diagram representation of differentially expressed miRNAs sharing and exclusive constitutively presented among samples. **(C–E)** Volcano plots of the −log_10_ p value vs the log_2_ miRNAs abundance comparisons between control and CVA2 infected hearts. MiRNAs outside the significance threshold lines with p-value < 0.05 were colored in blue, and miRNAs with p value < 0.05 and |log2 (fold change)| > 1.0 were colored in red. **(F–H)** Heatmaps of differentially expressed miRNAs.

**Figure 4:**
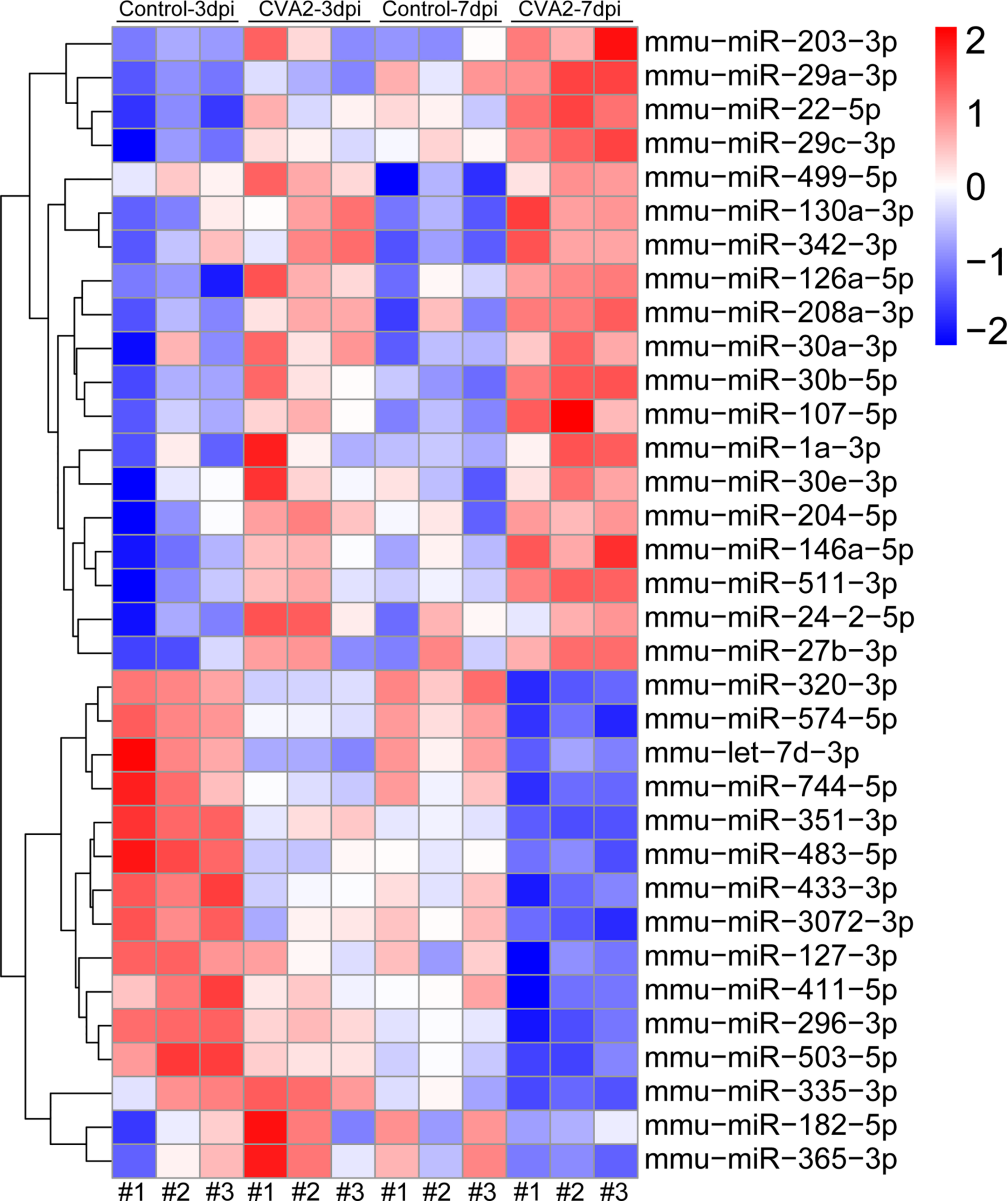
Visualization of differential co-expressed miRNAs in heart tissues of CVA2-infected mice. A total of 34 differential co-expressed miRNAs were identified in this study. The heatmap of these miRNAs was presented in the figure.

At 3 dpi, 7 dpi, and 3 dpi vs 7 dpi, a total of 87, 101, and 76 differential miRNAs (**Figures 3A, B** and **Tables S2****–****S4**) were identified, respectively. **Figures 3C–E** showed differential miRNAs with p-value <0.05 and the value of |log2 (fold change)| > 1 (red dots) of 3 comparison strategies. **Figures 3F–H** showed differential miRNAs expression patterns of 3 comparison strategies. Importantly, above 3 comparison strategies shared 34 differentially expressed miRNAs (**Figure 4** and **Table 2**), and 29 of them exhibited the same trend. Taken together, our data indicated alterations of miRNA profile in mice hearts after CVA2 infection.

**Table 2:** The differential co-expressed miRNAs.

miRNA	Fold change (3dpi)	Fold change (7 dpi)	Fold change (7 dpi/3dpi)
mmu-miR-203-3p	1.05	1.77	0.98
mmu-miR-29a-3p	0.42	0.88	1.68
mmu-miR-22-5p	0.89	0.99	0.98
mmu-miR-29c-3p	0.98	1.11	1.35
mmu-miR-499-5p	-0.65	0.96	0.40
mmu-miR-130a-3p	0.67	1.22	0.56
mmu-miR-342-3p	0.50	1.09	0.49
mmu-miR-126a-5p	0.76	0.80	0.48
mmu-miR-208a-3p	0.88	1.08	0.72
mmu-miR-30a-3p	0.52	0.80	0.43
mmu-miR-30b-5p	0.68	1.17	0.72
mmu-miR-107-5p	0.88	1.74	1.09
mmu-miR-1a-3p	0.59	0.95	0.58
mmu-miR-30e-3p	0.37	0.58	0.44
mmu-miR-204-5p	1.37	1.13	0.40
mmu-miR-146a-5p	1.42	1.69	1.16
mmu-miR-511-3p	1.10	1.40	1.05
mmu-miR-24-2-5p	0.65	0.46	0.29
mmu-miR-27b-3p	0.36	0.54	0.58
mmu-miR-320-3p	-0.68	-1.09	-0.26
mmu-miR-574-5p	-0.92	-1.41	-0.69
mmu-let-7d-3p	-0.86	-0.40	0.32
mmu-miR-744-5p	-1.08	-1.11	-0.50
mmu-miR-351-3p	-1.21	-1.05	-1.34
mmu-miR-483-5p	-1.95	-0.96	-0.61
mmu-miR-433-3p	-1.20	-0.99	-0.60
mmu-miR-3072-3p	-1.33	-1.42	-0.88
mmu-miR-127-3p	-0.52	-0.59	-0.59
mmu-miR-411-5p	-1.18	-0.50	-0.37
mmu-miR-296-3p	-0.77	-1.02	-1.52
mmu-miR-503-5p	-0.69	-0.46	-0.81
mmu-miR-335-3p	0.45	-0.43	-1.36
mmu-miR-182-5p	0.80	-0.94	-0.92
mmu-miR-365-3p	0.73	-0.53	-0.77

### Verification of Differentially Expressed MiRNAs by qPCR

**Figure 5:**
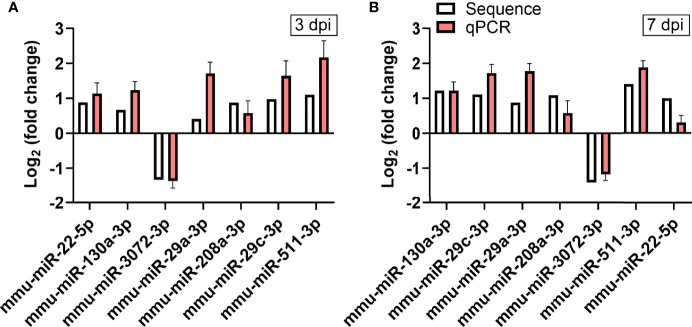
Verification of RNA-seq using qPCR. The expression of the 7 differentially expressed miRNAs identified by RNA-seq was analyzed using qPCR. **(A)** The 7 miRNAs changed at 3 dpi. **(B)** The 7 miRNAs changed at 7 dpi. The detection for each miRNA was repeated at least 3 times and the standard deviation was denoted as error bar.

To verify the validity of the RNA-seq-identified differential miRNAs, we performed qPCR on 7 of these miRNAs using the stem-loop technique. The fold-changes in gene expression of those miRNAs in heart tissues of CVA2-infected mice were calculated, using control mice for normalization (**Figures 5A, B**). Our data indicated that the qPCR data were consistent with the RNA-seq data on the basis of identifying up- and down-regulated miRNAs.

### Functional Enrichment of Differential MiRNAs

To study the regulation roles of the differential miRNAs in heart injury caused by CVA2, we conducted GO and KEGG enrichment analysis of differentially expressed miRNAs that had been identified at 3 dpi, 7 dpi, and 7 dpi vs 3 dpi.

**Figure 6:**
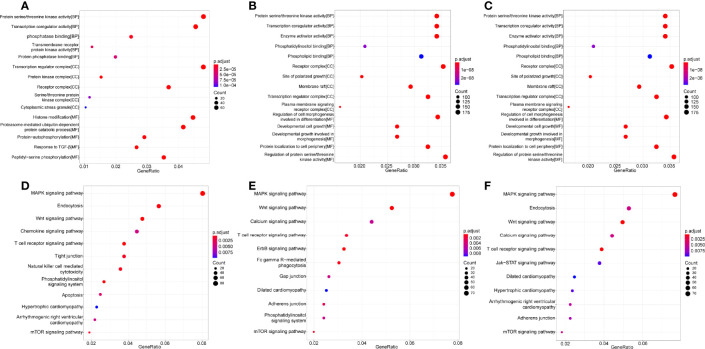
GO and KEGG enrichment analysis of significantly dysregulated miRNAs in heart tissues. **(A–C)** GO enrichment analysis of significantly dysregulated miRNAs in heart tissues. **(D–F)** KEGG enrichment analysis of significantly dysregulated miRNAs in heart tissues.

GO analysis includes three different aspects of biological process (BP), cellular component (CC) and molecular function (MF). Prediction terms with p-adjusted value < 0.05 were selected and ranked by enrichment score [−log2 (p-adjusted value)]. The 5 main GO terms were as follows: protein serine/threonine kinase activity (BP), transcription regulator complex (CC), regulation of protein serine/threoninekinase activity (MF), receptor complex (CC), regulation of cell morphogenesis involved in differentiation (MF) (**Figures 6A–C**).

The KEGG enrichment analysis was carried out to determine the principal functions of significantly dysregulated miRNAs. Prediction terms with p-adjusted value less than 0.05 were selected and ranked by enrichment score (−log2 (p-adjusted value)). KEGG analysis indicated that the differential miRNAs participated in 5 main pathways: MAPK signaling pathway, Wnt signaling pathway, Calcium signaling pathway, endocytosis, T cell receptor signaling pathway (**Figures 6D–F**). Other terms, like NK cell mediated cytotoxicity, Fc gamma R-mediated phagocytosis, Jak−STAT signaling pathway, apoptosis, tight junction, cardiomyopathy, mTOR signaling pathway were also enriched by multiple differentially expressed miRNAs (**Figures 6D–F**).

### Validation of MiRNA-mRNA Pairs

**Figure 7:**
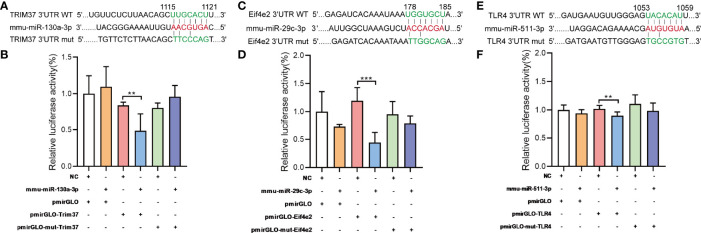
Target validations of differential miRNAs. **(A)** The series of miR-130a-3p combined with TRIM37; nucleotides in the seed region of pmirGLOTRIM37 and pmirGLOTRIM37-mut constructs are highlighted in red. **(B)** Luciferase reporter assays were carried out in HEK293 cells, using renilla luciferase as the endogenous control. Data were presented as mean ± SD and were generated from three independent experiments. **(C)** The series of miR-29c-3p combined with Eif4e2; nucleotides in the seed region of pmirGLOEif4e2 and pmirGLOEif4e2-mut constructs are highlighted in red. **(E)** The series of miR-511-3p combined with TLR4; nucleotides in the seed region of pmirGLOTLR4 and pmirGLOTLR4-mut constructs are highlighted in red. **(B, D, F)** Luciferase reporter assays were carried out in HEK293 cells, using renilla luciferase as the endogenous control. Data were presented as mean ± SD and were generated from three independent experiments (**p < 0.01, ***p < 0.001).

MiRNA controls target mRNA expression by imperfect base-pairing and binding the 3’UTR of target mRNA. TRIM37, Eif4e2, TLR4 are predicted to be one of the mmu-miR-130a-3p, mmu-miR-29c-3p, mmu-miR-511-3p target genes (**Figures 7A, C, E**), respectively. To further confirm the interaction between miRNAs and predicted mRNA targets, we performed a dual luciferase reporter assay using a vector encoding the 3’UTR of TRIM37 mRNA or Eif4e2 mRNA or TLR4 mRNA. We found that the relative luciferase activity was significantly reduced in the mmu-miR-130a-3p and TRIM37 transfections or mmu-miR-29c-3p and Eif4e2 transfections or mmu-miR-511-3p and TLR4 transfections compared to that of the control transfections (**Figures 7B, D, F**). Taken together, our results confirmed the positive interaction between mmu-miR-130a-3p and TRIM37, and mmu-miR-29c-3p and Eif4e2, and mmu-miR-511-3p and TLR4.

### Validation of Functional Enrichments

**Figure 8:**
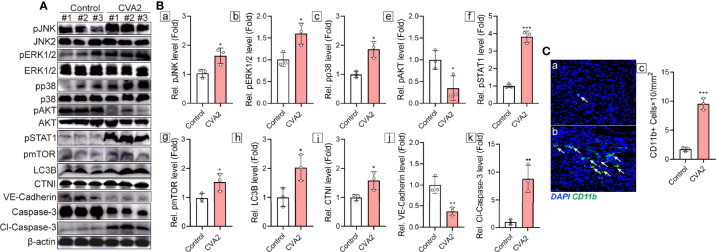
Validation of functional enrichments. At 7 dpi, CVA2-infected mice (n = 3) and controls (n = 3) were euthanized, and heart tissues were taken out for Western blotting (n = 3) and immunofluorescence staining (n = 3). **(A)** Western blotting analyses of pJNK, JNK2, pERK1/2, pp38, p38, pAKT, AKT, pSTAT1, pmTOR, LC3B, CTNI, VE-Cadherin, Caspase-3, Cl-Caspase-3. **(B)** Relative expression of pJNK (a), pERK1/2 (b), pp38 (c), pAKT (e), pSTAT1 (f), pmTOR (g), LC3B (h), CTNI (i), VE-Cadherin (j), Cl-Caspase-3 (k) were normalized by non-phosphorylated form or actin. **(C)** CD11b immunofluorescence staining of heart slices of control (a) and CVA2-infected mice (b). CD11b positive cells (C-c) were calculated by Image J. Data were presented as mean ± SD and were generated from three independent experiments (*p < 0.05, **p < 0.01, ***p < 0.001).

To further confirm the above enriched pathways and processes, Western blotting analysis was performed to detect MAPK signaling pathway (JNK, p38, ERK1/2), PI3K-Akt signaling pathway (Akt), Jak-STAT signaling pathway (STAT1), mTOR signaling pathway (mTOR. LC3B), myocardial injury (CTNI), apoptosis (Caspase-3), and tight junction (VE-Cadherin). As shown in **Figures 8A, B**, phosphorylation of JNK, p38, ERK1/2, STAT1, and mTOR in mice hearts was significantly increased after CVA2 infection, while phosphorylation of Akt was significantly reduced after CVA2 infection. The expression levels of CTNI, cleaved Caspase-3 in mice hearts from CVA2-infected mice were significantly elevated, compared to that of control mice. We also found the expression level of VE-Cadherin in mice hearts was significantly reduced after CVA2 infection. Additionally, we found the accumulation of NK cells in mice hearts from CVA2-infected mice using immunofluorescence staining (**Figure 8C**).

### MiRNA-mRNA Integrated Analysis

**Figure 9:**
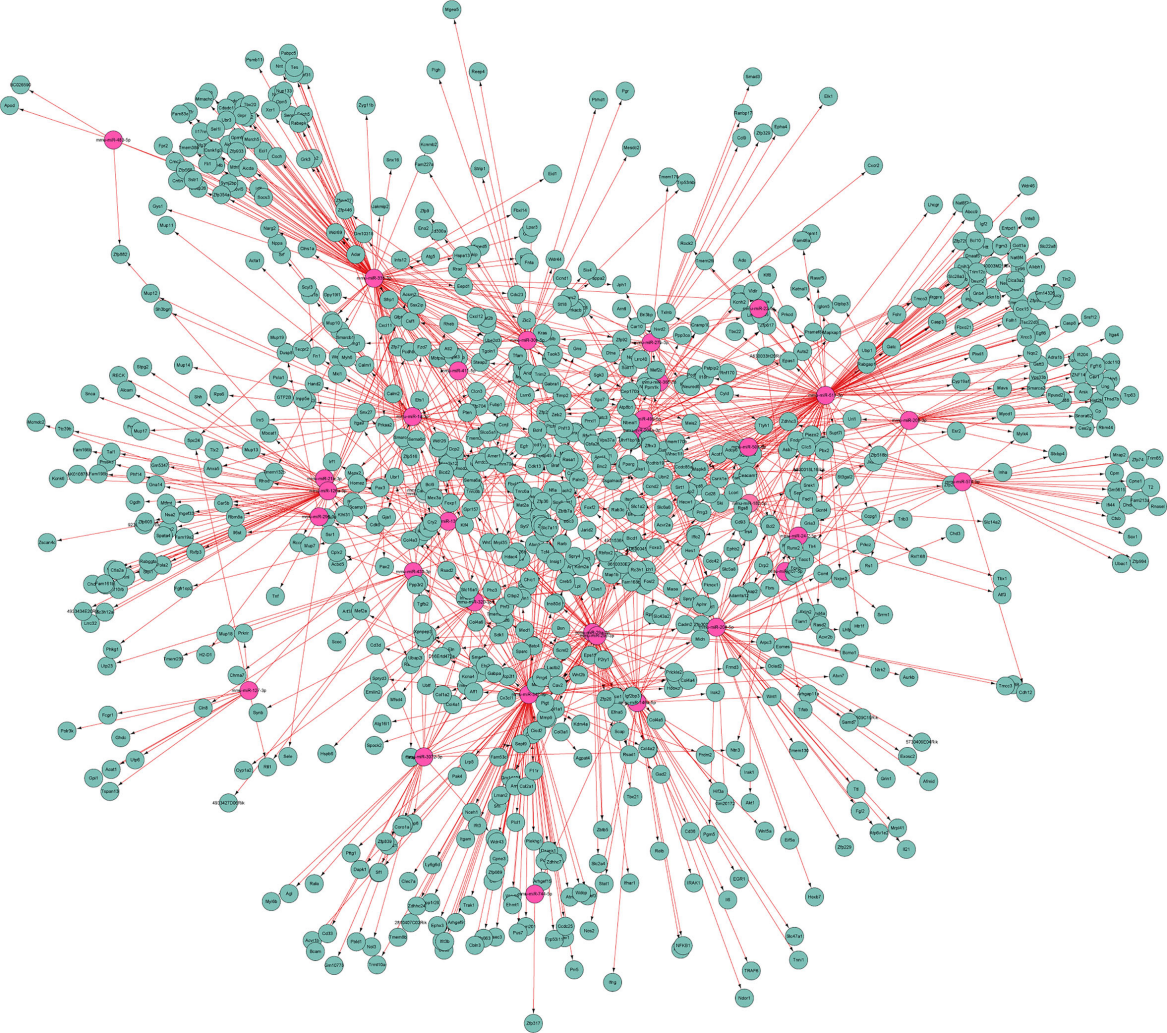
MiRNA-mRNA interaction network. It illustrates the predicted interactions of differential miRNAs with their targets. Red and green represent differential miRNAs and target genes, respectively.

To further uncover the relationship between differential miRNAs and predicted targets, the miRNA-mRNA integrated analysis was carried out. In this analysis, we selected 34 differentially co-expressed miRNAs and 983 target genes (**Figure 9**). The results showed that most of differential miRNAs had more than 5 target genes. For example, mmu-miR-511-3p was inversely correlated with 91 target genes, of which 14 target genes are associated with other differential miRNAs. More detailed data were shown in **Figure 9**.

## Discussion

CVA2 infection complicated with viral myocarditis has been reported worldwide (Bendig et al., 2001; Yip et al., 2013). Our previous study had demonstrated that CVA2 led to heart injury in a neonatal mouse model (Ji et al., 2021). However, the potential mechanisms of heart injury caused by CVA2 remain largely unknown. Emerging evidence had shown the important function of miRNAs in EV-induced innate immunity and there were numerous reports of miRNAs that are connected to Coxsackievirus infection (Zhu et al., 2021). To elucidate the molecular mechanism of heart injury caused by CVA2 infection, our report, for the first time, utilized CVA2 infected suckling mice heart for miRNA analysis to reveal the miRNAs that worked on mice heart injury.

In response to CVA2 infection, we identified 52 significantly dysregulated miRNAs at 3 dpi, and 73 differentially expressed miRNAs at 7 dpi. This difference might reflect the severity of infection, as can be seen in **Figure 1**, at 7 dpi, the mortality and physical condition of the mice were significantly severe than that at 3 dpi. We have also identified several important miRNAs in this study. Specifically, 34 miRNAs (**Table 2**) are commonly changed at 3 dpi, 7 dpi, and 7 dpi vs 3 dpi. Among them, miR-574-5p, 499-5p, 296-3p, 320-3p, 30e-3p, 30b-5p, 30a-3p, 29c-3p, 22-5p, 208a-3p, 24-2-5p participated in the differentiation and damage of cardiomyocytes, and heart diseases. It had been reported that miR-574-5p played a critical role in human cardiac fibroblasts (HCFs) myofibroblast differentiation (Cui et al., 2020). MiR-499-5p can protect the cardiomyocytes against apoptosis induced acute myocardial infarction (AMI) (Li et al., 2016). MiR-296-3p and miR-24-2-5p are important for embryonic stem (ES) cell (ESC) cardiac differentiation (Sun et al., 2011; Lee et al., 2016). MiR-320-3p can protect rat cardiomyocytes from ischemia/reperfusion (HR) injury through targeting Akt3 (Cao and Chai, 2020). MiR-30e-3p is involved in promoting cardiomyocyte autophagy and inhibiting apoptosis by regulating Egr-1 (Su et al., 2020). MiR-30b-5p exerts the suppression of fibrogenesis in Ang II-treated cardiac fibroblasts *via* targeting PTAFR (Zhao et al., 2020). MiR-30a-3p, miR-22-5p, and miR-208b-5p were considered as diagnostic biomarkers for heart diseases (Kakimoto et al., 2016; Wang et al., 2019; Ding et al., 2020). MiR-29c-3p can inhibit proliferation in rat primary cardiac fibroblasts. MiR-208a-3p aggravates autophagy in Ang II-induced H9c2 cardiomyoblasts (Wang et al., 2018). MiR-511-3p, 27b-3p, 433-3p, and 126a-5p were demonstrated to be related to antiviral immunity, viral replication, and infectious diseases. MiR-511-3p was reported to inhibit ZIKV replication *in vivo* (Tsetsarkin et al., 2020). MiR-27b-3p could enhance type I interferons (IFNs) expression and suppress virus replication (Duan et al., 2020). Serum miR-433-3pwas increased in patients with chronic hepatitis B infection (Tan et al., 2014). Xu et al. found that miR-126a-5p was involved in the hypoxia-induced neonatal pulmonary hypertension (Xu et al., 2017). Therefore, we speculated that miR-126a-5p might be related to neurogenic pulmonary edema in severe infections with HFMD (Peng et al., 2017).

Inflammatory abnormalities with excessive cytokine production, and the activation of immune cells contribute to HFMD severity (Zeng et al., 2013; Duan et al., 2014; Qin et al., 2019). Mitogen-Activated Protein Kinases (MAPKs) belong to a serine/threonine protein kinases family (Meena et al., 2019). MAPK activation induces the expression of multiple genes that together regulate the inflammatory response by enhancing pro-inflammatory cytokine production (Arthur and Ley, 2013). Activation of T lymphocytes is considered as a key event for an efficient response of the immune system, which requires T cell receptor (TCR) (Shah et al., 2021). Our previous study found T cell activation in EV71-infected mice (Jin et al., 2018). T cell receptor signaling pathway and its downstream MAPK signaling activation can lead to excessive the secretion of pro-inflammatory cytokines (Shah et al., 2021). Serum inflammatory cytokines (e.g. IL-6, TNF-α, IL-1β) and chemokines (e.g. CXCL10) were significantly elevated in severe patients with HFMD (Zeng et al., 2013; He et al., 2019). Our previous study also found that the concentrations of TNF-α, interleukin (IL)-1β, IL-6 and monocyte chemoattractant protein-1 (MCP-1) in heart tissues of CVA2-infected mice were significantly elevated (Ji et al., 2021). In this study, our histopathological examination also showed inflammatory cell infiltration. Together, our results suggest that inflammatory signaling pathways and T cell activation participate in CVA2-induced heart injury.

Virus infection can inflict significant damage on cardiomyocytes through direct injury and secondary immune reactions, leading to myocarditis (Yajima, 2011). Our results found that some of differentially expressed miRNAs were associated with cardiomyopathy. Ca^2+^/Calmodulin-Dependent Protein Kinase II (CaMKII) is a serine/threonine protein kinase that functions in cardiomyocytes, which plays an essential role in transcriptional activation associated with myocardial injury (Zhang and Brown, 2004). Cardiac troponin I (CTNI) is a key regulator in cardiac muscle contraction and relaxation, linking to Ca^2+^ signaling pathway (Layland et al., 2005). Our result confirmed the up-regulation of CTNI. Cellular cytotoxicity, is an important effector mechanism of the immune system to combat viral infections. Cytotoxic natural killer (NK) cells are the major mediators of this activity (Prager and Watzl, 2019). In the present study, we found the infiltration of NK cells (CD11b+) in mice hearts from CVA2-infected mice. Other than cytotoxic activity, NK cells activation is accompanied by production of pro-inflammatory cytokines. Hence, NK cells also have the potential to act in driving inflammation (Zitti and Bryceson, 2018).

Our results indicated that significantly dysregulated miRNAs were associated with viral entry into host cell (Endocytosis, Fc gamma R−mediated phagocytosis). Coxsackieviruses could infect cardiomyocytes, and due to extensive virus replication, a rapid cytolysis of these cells occurs (McManus et al., 1993). In the present study, myocardial fiber breakage, and myocardial interstitial edema were observed in heart slices of CVA2-infected mice. Our previous study demonstrated that CVA2 could infect cardiomyocytes in mice (Ji et al., 2021). Virus replication further results in apoptosis and autophagy in heart tissues of infected mice. Based on histopathological examination, we found apoptotic cells at the site of heart. Moreover, some significantly dysregulated miRNAs were found to be related to the process of apoptosis in this study. Our results suggest that CVA2 infection triggers autophagy (mTOR signaling pathway) and apoptosis (Cl-Caspase-3). Autophagy, a conserved catabolic process, plays an immensely important role in heart diseases (Saha et al., 2018). Recently, more and more attentions have been paid to the mechanisms of mTOR in autophagy regulation (Kim and Guan, 2015). KEGG analysis indicated that some differentially expressed miRNAs participated in Jak-STAT pathway. In response to virus infection, antiviral cytokines (IFN-α/β) were produced by host cells upon viral detection by pathogen recognition receptors. IFN-α/β embarks upon a complex downstream signaling cascade called the JAK/STAT pathway. This pathway leads to the expression of hundreds of effector genes known as interferon stimulated genes (ISGs). ISGs are the basis for an elaborate effector mechanism and ultimately, the clearance of viral infection (Raftery and Stevenson, 2017). Our experimental data demonstrated the activation of Jak-STAT signaling pathway during CVA2 infection.

Accumulating evidence demonstrate that Wnt signaling pathway is activated during the pathogenesis of many heart diseases (Foulquier et al., 2018). Our data indicated that multiple significantly dysregulated miRNAs were enriched in Wnt signaling pathway. It had been reported that up-regulated miR-126 promoted CVB3 replication by mediating cross-talk of ERK1/2 and Wnt/β-catenin signal pathways (Ye et al., 2013). Tight junctions enable epithelial cells to form physical barriers, acting as an innate immune mechanism against viral infection (Zeisel et al., 2019). Therefore, the disruption of epithelial tight junctions promotes the infection and spread of the virus. VE-cadherin is a component of endothelial cell-to-cell adherens junctions, and it plays an important role in the maintenance of vascular integrity (Giannotta et al., 2013). In this study, we found that the expression of VE-cadherin was reduced in mice hearts from CVA2-infected mice.

**Figure 10:**
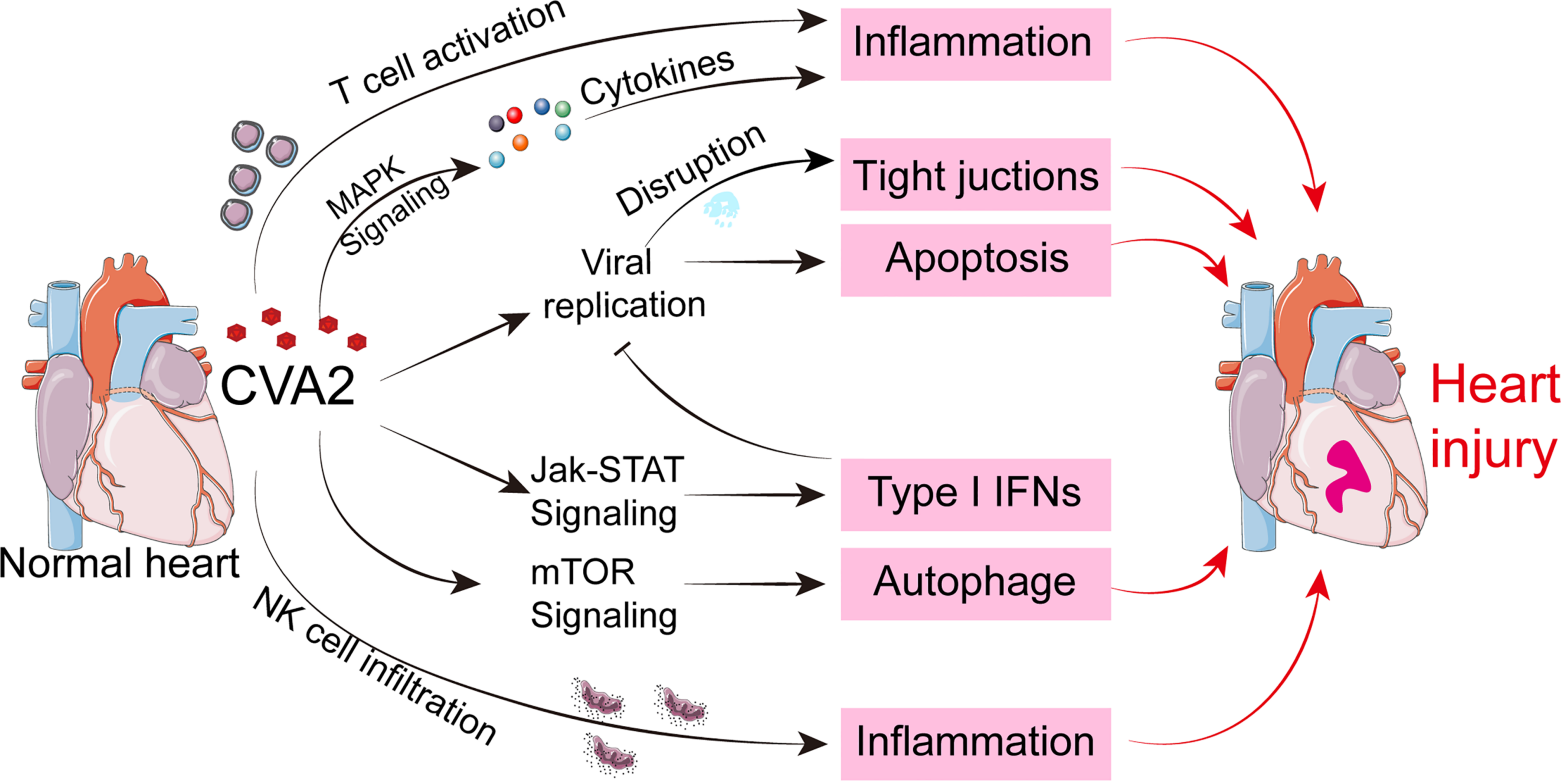
A hypothetical schematic of the mechanisms of heart injury caused by CVA2.

In summary, the present study revealed unique sets of miRNAs during heart injury caused by CVA2 infection and their potential roles were predicted by bioinformatics softwares. By verifying the results of bioinformatics analysis, our results suggest that inflammatory responses, T cell activation, apoptosis, autophagy, antiviral immunity, and NK cell infiltration, and the disruption of tight junctions are involved in the pathogenesis of heart injury caused by CVA2 (**Figure 10**), which explain the major clinical manifestation of hyperinflammatory status of severe patients with HFMD. These findings provided insights into the pathogenic mechanisms of CVA2 infection and further understanding of HFMD-associated viral myocarditis. As a word of caution, however, the results presented here are based on miRNA targets prediction and pathway analysis, more experiments are required to confirm the data in order to find efficient therapeutic strategies for heart injury caused by CVA2.

## Data Availability Statement

The original contributions presented in the study are publicly available in the National Genomics Data Center (NGDC), part of the China National Center for Bioinformation (CNCB) under accession number CRA004886.

## Ethics Statement

The animal study was reviewed and approved by The Life Science Ethics Review Committee of Zhengzhou University.

## Author Contributions

Conceptualization, YJ and ZW. Data curation, ZW, WJ, and YJ. Formal analysis, ZW, SZ, WJ, JQ, and YH. Funding acquisition, YJ. Investigation, ZW, WJ, DL, PZ, and RL. Methodology, YJ, RL, and ZW. Project administration, YJ. Resources, ZW, WJ, and SZ. Software, SZ, JQ, and YH. Supervision, YJ. Validation, DL, SZ, and JQ. Visualization, PZ and YH. Writing – original draft, ZW and YJ. Writing – review and editing, YJ, ZW, WJ, PZ, RL, DL, JQ, SZ, and YH. All authors contributed to the article and approved the submitted version.

## Funding

This research was funded by the National Natural Science Foundation of China (NO.82002147); China Postdoctoral Science Foundation (NO.2019M662543); Key Scientific Research Project of Henan Institution of Higher Education (NO.21A310026).

## Conflict of Interest

The authors declare that the research was conducted in the absence of any commercial or financial relationships that could be construed as a potential conflict of interest.

## Publisher’s Note

All claims expressed in this article are solely those of the authors and do not necessarily represent those of their affiliated organizations, or those of the publisher, the editors and the reviewers. Any product that may be evaluated in this article, or claim that may be made by its manufacturer, is not guaranteed or endorsed by the publisher.

## Supplementary Material

Supplementary Table 1 All detected miRNAs with TPM > 1.

Supplementary Table 2 Differentially expressed miRNAs at 3 dpi.

Supplementary Table 3 Differentially expressed miRNAs at 7 dpi

Supplementary Table 4 Differentially expressed miRNAs (7 dpi vs 3 dpi).
